# Case of Excessive Hyperæsthesia

**Published:** 1846-01

**Authors:** Henry Haines Fox

**Affiliations:** Columbia, Pennsylvania, Student of Medicine in Jefferson Medical College


*Case of Excessive Hyperæsthesia.* By HENRY HAINES FOX, of Columbia, Pennsylvania, Student of Medicine in Jefferson Medical College. Communicated in a letter to PROFESSOR DUNGLISON.

[The following case is not unique, but it is sufficiently strange, and has excited great interest in all who have seen it. We have met with one or two instances in which the power of the voluntary muscles appeared to be lost; and, at the same time, great exaltation of certain of the senses existed, which passed wholly away, and left the patient well. One of these occurred in a young lady whose friends consulted many of the most distinguished members of the Faculty; their prescriptions were, however, of no avail. The morbid condition ultimately disappeared during the new evolutions that occurred at puberty.]

*Philadelphia,* Nov. 28th, 1845.

DEAR SIR,—The case of general paralysis followed by hyperacusis, in a boy aged 11 years and 9 months, at the time he was first attacked, and concerning which I consulted you last winter, has evidently improved in many respects under the treatment recommended by you; which was, as you will recollect, to avoid as much as possible everything that would tend to irritate, or aggravate him in any respect, mentally or corporeally, and to trust to the recuperative powers of the system.

As the case is a singular one, and may interest you, I will give you a brief history of it from the commencement. The first thing that attracted the attention of his parents was a hard rough cough, which occurred in January, 1844. He had, however, complained occasionally of wandering pains in his shoulders, with slight weakness of his limbs upon rising from bed in the morning, for some months previous to that time, but these soon passed away. The cough became gradually worse, accompanied with pains and soreness in his teeth, mouth and throat; until the latter part of February, the coughing was almost incessant especially in the day time, although not attended with any expectoration. At this time a physician was called in who pronounced the disease to be inflammation of the lungs, and treated it accordingly; he did not, however, order the patient to lose blood. After the application of the second blister to his breast, the cough left him entirely and the physician ceased to visit him. It was not long, however, before he began to complain again of his jaws and throat, so that it was with difficulty that they could prevail on him to take nourishment, from the pain and difficulty attending deglutition. From this time, he began to lose strength, and became very costive, having no evacuation for several days; but by repeated injections they succeeded in procuring one. After the first enema he wholly lost the use of his limbs, and has not been able to help himself in the least up to the present period. After the second enema he lost all control over his eyelids, for several days, but it has since partially returned to him. If requested to move them when open, they almost invariably closed in an instant: this condition continued for a period of several months, but he gradually recovered, so that now they are under the influence of the will as well as before his illness. About the first of April, 1844, he began to complain of his head, whenever he was moved, and in a short time his parents were unable to move him or change his position in bed. Owing to his weakened and prostrated condition, they have been unable to ascertain whether there is tenderness along the spine, as the least movement or change of position is attended with the most alarming symptoms, the last time his bedclothes were changed he remained senseless —perfectly unconscious of every thing—for a period of two hours. It was in April that he began to complain of noise affecting him; and the hyperæsthesia of the organ of hearing soon became so great, that the barking of a dog outside the house would throw him into an insensible state for minutes. Although his ears are well filled with cotton, to prevent as much as possible the immediate contact of noise with the supersensitive organ, such was his impressible condition that his father was compelled to relinquish farming for several months, being unable to thrash his grain, or drive his team past the house.

In the latter part of summer, he experienced stitches in the posterior part of his head, which were followed by pain in the back, shooting up to the head; but these finally left him, so that at the present time he does not complain of any. His reason seems to have become impaired with the increase of the malady, especially on some points, though not on all,—often conversing for hours without showing any impairment of the mental faculties whatever. His memory has remained perfect throughout the illness, he recollects apparently every thing that has occurred, but his temper is greatly changed; he often breaks out into violent rage, and at the same time makes use of language which he never did before his sickness. His shyness, or dread of strangers commenced about the time he lost the use of his limbs. He cannot be prevailed upon by any of the family to permit many of his near relatives to see him, but above all he objects to physicians; for whom he has the greatest dread imaginable. For more than a year anorexia was great, so that it was often difficult to prevail on him to take the least quantity of nourishment; consequently he became greatly emaciated; of late, however, his appetite has improved greatly—he has become more fleshy, and has evidently grown within the last nine months. He has had no medical treatment since his first attack: upon this point his father—in a letter (dated Feb. 19th, 1845)—speaks thus—"I sincerely believe that it was impossible to have resorted to any active means since last May, owing to his great prostration, and utter abhorrence of all physicians. I have been expecting every day would be his last for some months."

Since last April the supersensitiveness of the auditory nerve, has been gradually diminishing, until it has become nearly natural; and he now seldom complains of noise disturbing him, unless it is very loud. His appetite has returned, so that he takes a good share of nutritious aliment consequently; his nutrition has greatly improved and he has evidently grown in stature within the last nine months. Although still unable to exert any control over his limbs or body when awake, it has been observed, of late, that he does change their position in his sleep. These are among the more marked changes which have occurred since last winter.

I am, dear sir, your humble and
obedient servant,
HENRY HAINES FOX.

PROF. DUNGLISON.

